# Negotiating Gender Norms to Support Men in Psychological Distress

**DOI:** 10.1177/1557988317733093

**Published:** 2017-10-11

**Authors:** Aisling Keohane, Noel Richardson

**Affiliations:** 1 Institute of Technology Carlow—Science and Health, Carlow, Ireland

**Keywords:** suicide prevention, gender, help-seeking, help-giving, gatekeepers, men’s health

## Abstract

Underpinning a general pattern of higher suicide rates in men is the assumption that men do not ask for help or utilize the health-care system during times of psychological distress. There has been a failure to grapple with the dynamic of when, how and from whom men might ask for help during times of psychological distress, and what key barriers or enabling factors are likely to influence potential help-givers’ capacity or willingness to offer help to men in psychological distress. The aim of this study was to investigate how masculine norms impact men’s help-seeking as well as care givers’ behaviors and willingness to support men in need of psychological help or perceived to be at risk of suicide. Focus groups (*n* = 13) were used with “high-risk suicide” groups of men and community gatekeepers. The principles of grounded theory were used for data analysis. Three themes emerged: “negotiating ways to ask for, offer and accept help without compromising masculinity”; “making and sustaining contact with men in psychological distress”; and “navigating roles responsibilities and boundaries to support men in psychological distress.” Approaches to suicide prevention need to take account of how masculine norms shape men’s willingness to ask for and accept help during times of psychological distress as well as care givers willingness to offer help. The findings address a gap in the literature by looking beyond men’s help-seeking as a passive, one dimensional construct, to a more dynamic triad of help-seeking/giving/taking behaviors that are embedded in the sociocultural context of men’s lives.

In most western countries, mortality from suicide is typically three to four times higher in males, yet rates of suicide ideation and deliberate self-harm are higher in females (World Health Organization, 2012). It is also well established that while women have higher levels of depression and anxiety (or internalizing disorders), men have higher levels of substance abuse and antisocial disorders (or externalizing disorders) (European Commission, 2011). In attempting to account for this “gender paradox” (Galdas, Cheater, & Marshall, 2005), a number of intersecting factors have been identified that mediate the relationship between gender and suicide (Canetto & Sakinofsky, 1998; Payne, Swami, & Stanistreet, 2008). These include methods used (men typically use more violent methods) (Varnik et al., 2009) the presence of underlying mental health issues (which often remain undiagnosed in men) (Esposito, 2002), alcohol and substance use (which are higher in men) (Miller & Taylor, 2005), help-seeking and use of health care (men tend to be reticent to seek help or access services when in psychological distress) (Johnson, Oliffe, Kelly, Galdas, & Ogrodniczuk, 2012), sexuality (shame and stigma experienced by gay men) (Bybee, Sullivan, Zielonka, & Moes, 2009), and social and community factors (including socioeconomic status, employment status, marital status, societal change, and changing gender roles) (Cleary & Brannick, 2007).

Approaches to suicide prevention are increasingly seeking to account for contextual, systemic, and sociocultural risk and protective factors and determinants—what Ashfield, MacDonald, and Smith (2017) refer to as “situational suicide prevention”—that revolve around the real world of individuals’ lived experience. In Ireland (Health Service Executive, 2016; Richardson, 2013) and indeed globally (World Health Organization, 2012), there have been increasing calls for a more explicit focus on gender in relation to approaches to suicide prevention.

One of the key factors that has been attributed to men’s higher suicide rates is the assumption that men do not ask for help or utilize the health-care system during times of psychological distress (Grace, Richardson, & Carroll, 2016; White et al., 2011). A number of reasons and explanations have been put forward for this. Discourses on masculinity and help-seeking typically frame more traditional masculine practices with being stoic, emotionally restrictive, and denial of vulnerability (Addis & Mahalik, 2003).

With seeking help during times of psychological stress being seen as “weak” (Oliffe & Phillips, 2008), many men suffer in silence out of fear of rejection from their peers (Connell, 2005). Indeed, the notion of a “double jeopardy” (Levant, Hall, Williams, & Hasan, 2009; Houle, Mishara, & Chagnon, 2008) describes men’s sense of shame in asking for help as compounding their “failure” to manage their own problems. It has been proposed that suicide may be perceived by these men as a way to regain control over their lives and to end their suffering on their own terms. Paradoxically, in seeking to re-establish a sense of control during times of psychological distress, and as an alternative to asking for help or accessing health care, many men revert to alcohol and other substances to relieve emotional and mental pain (Oliffe, Orgodniczuk, Bottorff, Johnson, & Hoyak, 2012).

In attempting to cope with psychological distress, Brownhill, Wilhelm, Barclay, and Schmied (2005) described how men choose to “act in” by blocking out problems, often resulting in “acting out” through risk-taking and aggressive behaviors. Studies that have reported more “positive” or adaptive coping behaviors used by men have typically focused on two key strategies—“practical solutions” and “problem solving” (Whittle et al., 2015). Addis (2011) similarly reported that men choose terms like “doing,” “taking,” and “deciding” in relation to their own help-seeking for depression, as opposed to terms like “being in therapy” or “receiving help.” Clearly therefore, gender influences men’s willingness or propensity to ask for help or access health services during times of psychological stress, with many men reverting to alternative “coping” strategies.

The embodiment of depression that may be more common among men—in terms of anger, isolation and autonomy around self-management practices, and risky self-care practices such as substance abuse—can lead to symptoms of depression being interpreted as expressions of masculine ideals and not symptoms of extreme distress (Oliffe et al., 2012). It has been proposed that current screening tools lack sufficient sensitivity and are better suited to detecting depression in women (Rice, Fallon, Aucote, & Möller-Leimkühler, 2013; Oliffe & Phillips, 2008). Indeed, screening tools that adopt labels such as “anxiety” or “depression,” may lead to underreporting in men to avoid being diagnosed with a stigmatizing mental health label thus having consequences for the likelihood of men seeking help (Milewa, Calnan, Almond, & Hunter, 2000). As a result, depression often remains undetected and untreated in men (Borowsky et al., 2000), particularly among groups of men who identify with more hegemonic masculine norms (Busfield, 2011).

It is well documented that help-seeking is more likely when the help-seeker trusts and has confidence in the person from whom he/she is seeking help. Berger, Addis, Green, Mackowiak, and Goldberg (2012) reported that having a genuine connection and mutual understanding with a person offering help was a critical factor in men’s willingness to seek help and to talk openly about their depression. It has been proposed that men prefer therapies that provide an open and noncontrolling atmosphere, free from judgment, where men can receive support, add and participate in any decisions made, therefore maintaining their sense of autonomy and self-control (Kivari, Oliffe, Borgen, & Westwood, 2016; Strike, Rhodes, Bergmans, & Links, 2006). Seidler, Dawes, Rice, Oliffe, and Dhillon (2016) demonstrated how tailoring and targeting health interventions towards men may result in an increase in men’s service engagement as well as the efficacy of treatments.

A great deal of literature on men’s health and male suicide has focused on help-seeking, with comparatively less focus on help-giving or what motivates individuals to participate in helping behaviors within their communities (Bastiaensens et al., 2014; Eagly, 2009). An Australian study challenged what it saw as the disproportionate focus on men’s help-seeking and called for a greater focus on moving beyond simplistic representations of male suicide and help-seeking and instead focusing on the complex interplay between help-seeking and health services (River, 2016). Early suicide prevention research identified two main categories of help-givers (Ramsay, Cooke, & Lang, 1990). “Emergent” help-givers (such as clergy, coaches, teachers, friends, or family) more typically have not been formally trained to intervene with those at risk of suicide. “Designated” help-givers comprise professionals who have been formally trained to intervene, (such as doctors, nurses, psychologists, and social workers). While emergent groups may be more strategically positioned to support those at risk of suicide and be the help-seeker’s preferred source of support (Cerel, Padgett, Robbins, & Kaminer, 2012; Cross, Matthieu, Cerel, & Knox, 2007), individuals within the emergent groups typically feel unskilled and under qualified in relation to identifying warning signs of somebody at risk of suicide, and anxious about how to intervene appropriately (Berger et al., 2012; Yap & Jorm, 2011).

Against a backdrop of poor levels of mental health literacy within communities there is often difficulty in identifying those who are experiencing psychological distress or likely to be at risk of suicide (‘U.S. Department of Health and Human Services, 2001). This leads many to feel unskilled and under qualified (Yap & Jorm, 2011). Limited knowledge within communities on the warning signs of suicide and steps to take to seek help and give help have been identified in numerous studies (Maine, Shute, & Martin, 2001; Owens et al., 2011). This highlights the importance of gatekeeper training that enables trainees to identify those at risk, assess the level of risk, manage the situation accordingly, and ultimately to increase the number of interventions with those in distress (Gould & Kramer, 2001; Isaac et al., 2009).

Previous studies have identified a gendered dimension to help-giving. For example, Eagly (2009) reported how men tend to offer “heroic help” (e.g., in emergency situations), “interventionist help” (e.g., to strangers encountering accidents), and “chivalrous help” (e.g., to women). Women, on the other hand, tend to extend care to children and elderly relatives, offer sensitive emotional support to spouses and friends, and relational support to workplace peers and subordinates. More typically, girls compared to boys, appear to have higher behavioral intentions to comfort individuals in distress, offer them advice and defend their entitlement to be distressed (Bastiaensens et al., 2014). To account for sex differences in helping, it is important to consider ways in which helping is influenced and sustained by social norms ascribed to particular roles in society.

In summary therefore, much of the literature to date that has focused on men’s health and suicide has had a narrow focus on men’s (lack of) help-seeking, with a particular emphasis on the relationship between gender and men’s maladaptive coping mechanisms during times of psychological distress. There has been a paucity of suicide prevention research that has sought to investigate the dynamic of when, how and from whom men may be more open to asking for help during times of psychological distress, and what key barriers or enabling factors are likely to influence potential help-givers’ capacity or willingness to offer help to men in psychological distress.The purpose of this study was to inform a gendered approach to suicide prevention efforts in Ireland, with a particular focus on investigating the contexts in which men ask for and receive support during times of psychological distress. There was a particular focus on exploring ways in which masculine norms impact men’s help-seeking as well as care givers behaviors and willingness to support men in need of psychological help or perceived to be at risk of suicide. The study was commissioned by the National Office for Suicide Prevention in Ireland.

## Method

### Data Collection

Ethical approval was granted by the Institute of Technology Carlow’s Ethics Committee. Focus groups (*n* = 13) were chosen as the main research tool for this study and snowball sampling was used to recruit participants. Participants (*n* = 69) were selected on the basis of specific sociodemographic characteristics identified in the wider literature as being associated with either (a) a higher risk of suicide (“HRS”) or (b) with a community gatekeeper (“CG”) role. In the context of the former, participants were selected based on assigned risk via their demographics rather than having any known mental health challenges. It should be acknowledged that while participants were typically recruited on the basis of one specific characteristic (e.g., being a “young man” or a “teacher”), many might have straddled multiple characteristics. A breakdown of the participants can be seen in Table 1.

**Table 1:** Biographical details of the focus group participants.

**Focus group participants (*n* = 69)**
**Higher risk of suicide (*n* = 34)**
**Lesbian, gay, bisexual, and transgender student group (*n* = 9)**
Female (*n* = 4); male (*n* = 5); age range (17–26)
**Travellers (*n* = 4)**
Female (*n* = 4); male (*n* = 0); age range (20–50+)
**Young men (*n* = 7)**
Female (*n* = 0); male (*n* = 7); age range (19–35)
**Unemployed (*n* = 6)**
Female (*n* = 0); male (*n* = 6); age range (27–50+)
**Men with a history of substance misuse (*n* = 6)**
Female (*n* = 0); male (*n* = 6); age range (27–35)
**Farmers (*n* =2)**
Female (*n* = 0); male (*n* = 2); age range (35–55)
**Community gatekeepers (*n* = 35)**
**Farm officials (*n* = 5)**
Female (*n* =1); male (*n* = 4); age range (35–55)
**Men’s health professionals (*n* = 6)**
Female (*n* = 1); male (*n* = 5); age range (30–55)
**Teachers (*n* = 5)**
Female (*n* = 5); male (*n* = 0); age range (25–35)
**Working with** T**ravel**l**ers (*n* = 2)**
Female (*n* = 1); male (*n* = 1); age range (35–50)
**Public health nurses and physiotherapist (*n* = 6)**
Female (*n* = 6); male (*n* = 0); age range (35–55+); physiotherapist (*n* = 1); nurse (*n* = 5)
**Religious group (*n* = 6)**
Female (*n* = 2); male (*n* = 4); age range (27–55+); catholic priest (*n* = 2); nun (*n* = 2); prayer group organizer (*n* = 1); counselor/prayer retreat organizer (1)
**Coaches (*n* = 5)**
Female (*n* = 0); male (*n* = 5); age range (27–32); athlete/coach (*n* = 3); school coach (*n* = 1); professional athlete (*n* = 1)

This sampling strategy was adopted to maximize a diverse range of perspectives around the central research question. While the focus of discussions was on the wider context of men’s psychological suffering more broadly and how men ask for and are offered help during such times, participants were encouraged to reflect at all times on their personal experiences and reflections on the questions that were posed, rather than speaking from the perspective of a HRS or CG representative. Participants were not made aware of their recruitment as HRS or CG but instead encouraged to move freely between roles and between different experiences of asking for (or not) and offering (or being offered) help during times of psychological distress. Written informed consent as well as permission for the discussions to be audio taped was sought from participants in advance. In keeping with a grounded theory approach (Corbin & Strauss, 1990), the topic guide was constantly updated in response to emerging findings to facilitate deeper investigation of emerging trends in the data.

The topic guide focused on three main areas: (a) meaning of psychological distress and personal triggers of psychological distress, (b) help-seeking (personal experiences), coping behaviors and identifiable sources of help, and (c) help-giving (personal experiences & meaning of giving help to someone in psychological distress), motivation for giving help, supports for offering someone help and different situations of help-giving. Each participant was encouraged to reflect on personal experiences of psychological distress that incorporated a broad range of psychological suffering. It was important to keep the term “psychological distress” as open as possible to be inclusive of participants’ diverse experiences as well as the potential of psychological distress to be associated with suicidal ideation or suicidal behavior. Field notes and personal journaling were used to record all observations and to contextualize the content of the interviews during the transcription and data analysis phases.

Appropriate support measures were put in place for researchers to debrief following data collection as well as for participants who may have felt distressed as a result of issues brought up in the focus groups. The use of reflexive memos allowed the principal investigator (PI) to elaborate on coding sessions as well as to reflect back on coding notes. It ensured that the coding of successive focus groups was informed by the experience and learning from previous groups (Strauss & Corbin, 1994).

### Data Analysis

All audio data were transcribed verbatim. Pseudonyms were used to protect participants’ anonymity. The initial transcripts were independently coded by both authors, which resulted in the development of a list of codes and emerging key concepts. Any field notes that were collected were added to support codes. Codes were further developed and refined using constant comparative analysis. This analysis involved the comparison of code lists, negotiating different interpretations and refining codes to agree a cumulative or “master” code list. At a later stage of the analysis, selective coding occurred, that is, codes were unified around a core category. This led to the emergence of both descriptive and explanatory categories, comprised of raw units of information from the transcripts and field notes. Relationships between categories were developed and refined over the course of the analytical process, leading to the development of themes and sub themes (Corbin & Strauss, 1990).

In the context of reporting findings “all” participants refers to 100% of participants, “most” refers to >90%, “the majority of” refers to 60–90%, “many” refers to 30–60%, and “some” or “a minority” refers to <30%.

## Results

The current findings revealed a dynamic triad of help-seeking, help-giving, and help-taking behaviors that intersected in multiple and complex ways and that were shaped by the sociocultural context of participants’ lives. Three overarching themes emerged: “negotiating ways to ask for, offer, and accept help without compromising masculinity”; “making and sustaining contact with men in psychological stresses”; and “navigating roles responsibilities and boundaries to support men in psychological stresses.” These themes will be explored in depth in this section.

### “Negotiating Ways to Ask for, Offer, and Accept Help Without Compromising Masculinity”

In seeking help, offering help, or accepting the offer of help, consideration invariably had to be given to potential implications, challenges or threats to masculinity. Being in psychological distress was seen as posing many challenges and threats to a man’s masculine identity, not least being a loss of power, control and autonomy. It was felt that cultural norms for men to withhold emotions and to maintain a stiff upper lip in the face of adversity, were still very prevalent in Irish society and were magnified for men in more traditionally masculine domains such as sporting or workplace settings:

**“…where I worked for 30 years [army] was probably another reason why I didn’t talk; because it was ‘a crime’, and you are not supposed to be depressed you’re not supposed to cry.” (Damon, Religious Group)**

Not surprisingly, acknowledging or “admitting” the problem to oneself was seen as the most difficult first step for many men. HRS groups, in particular, reflected upon the uncertainty and confusion that typically engulfed men during times of psychological distress, which they felt further compromised their decision-making and willingness to seek or indeed to accept help. Thus, paradoxically, it was felt that prevailing gender norms meant that the most distressing of the problems for men were often the ones least spoken about. The notion of a “double burden” referred to the perception that among more vulnerable men in particular, the act of seeking help and coming forward as being vulnerable or psychologically distressed, might further undermine an already fragile masculine identity. Mark (Young Men) describes how the prospect of opening up when feeling down potentially added insult to injury for a man in receipt of unemployment benefit (“on the dole”), as it compounded that man’s loss of pride and was a further betrayal of masculinity:

**“…all of a sudden they’re home and in the dole queue; it’s the pride ya know and embarrassment of feeling that way…like mentally wise, if you’re feeling down or whatever, I think it’s just another case of asking for help.” (Mark, Young Men)**

It was felt that men asking for, and accepting the offer of help, was legitimized in circumstances where personal problems were perceived to have reached a certain threshold of distress. It was also noteworthy that many of CG groups, in particular, felt that they would be more proactive in giving help in cases where someone had reached a threshold of psychological distress. This, they reasoned, provided a greater degree of certainty that help was warranted and thus reduced the potential risk of offering help where help was not wanted. Indeed, even in circumstances where such a threshold was reached, some participants, somewhat paradoxically, felt that before giving help, a volatile or crisis situation needed to be calmed before one could approach with an offer of help. It was felt there wasn’t much that could be said until “after the storm” (Jenny, Teacher) when the help seeker would be more receptive to taking any offer of help.

Confiding in someone was contingent on having trust and confidence in that person:

**“If you go and talk to someone…you’d have to fully trust that they’re not going to go out on the street telling people about you.” (Mark, Coach).**

The question of “who” one should trust or have confidence in was contentious however the vast majority of participants felt that family or friends were best placed and able to offer sufficient support during times of psychological distress. Older HRS participants, in particular, spoke about the pivotal role of female spouses in being alert to and supporting them through times of psychological distress or crisis. Others felt drawn to what they regarded as the more objective, professional and at arms-length support of appropriate “experts,” in whom there was also an expectation of increased objectivity and confidentiality. CG participants, in particular, highlighted an underlying sense of frustration and despair, in that even if they did “their job” as help-givers, their efforts were still likely to be thwarted by professional services and supports that were seen as largely ineffective:

**“There’s no point in us doing everything right if we can’t get our hands on the support services.” (Fiona, Working with Travellers)**

Thus, in the absence of adequate backing from support services, the consensus among CG participants was that, as potential help-givers, they might think twice about intervening to help.

Reflecting on approaches to giving help, the majority of participants felt that, in offering help, it was essential to provide support in a way that would not compromise the man’s adherence to masculine norms around self-reliance or emotional control. While the majority of participants felt that “men don’t talk,” many highlighted that men do seek support and offer help but in ways that are not traditionally seen as emotionally supportive. There was repeated reference to the nuances and subtleties of men’s use of language in relation to mental health and to the importance of meeting men “at their level” rather than imposing a right or a wrong way of communicating about mental health issues:

**“…some people will use evasive language or will talk around it… and maybe we should respect that rather than say there’s a certain way of talking about suicide…” (James, Men’s Health Professional)**

It was felt that, more typically, men preferred more practical or “blunt” forms of support that enabled them to retain a sense of (masculine) “normality.” This fear of being treated differently, “pampered” or not being seen as “normal” following disclosure of a reason for being psychologically distressed emerged as a key barrier to help-seeking for men:

**“You wouldn’t want people treating you differently, you still want the lads to give ya a bit of a slagging; you don’t want lads changing in that way…” (Pat, Coach)**

In circumstances where more masculine forms of help were offered, such as Pat’s blunt Irish expression of support **“cop on to yourself” (Pat, Coach)**, meaning to move on from something, was seen as both an acceptable means of offering and accepting support.

### “Making and Sustaining Contact With Men in Psychological Distress”

The theme “making and sustaining contact with men in psychological distress” emerged as having a key bearing on approaches to asking for, offering and accepting help. Participants spoke about the critical aspects of men’s lives that kept them “in contact,” including the connection and belonging associated with daily rituals and routines of family, community, work and sport. When these were disrupted – such as through relationship break-up, unemployment or retirement—men’s sense of autonomy, place, identity and belonging were undermined which potentially left them feeling bereft and “disconnected.” In this context, many reflected upon what they felt had been a more endemic erosion of family and community networks and support systems within Irish society.

This was seen as having impacted negatively on the sense of connection, and togetherness that were identified as the traditional hallmarks and fabric of Irish communities, particularly rural and Traveller communities. This erosion of networks and support systems emerged for participants for varying reasons. For Travellers, it was felt that Traveller customs and traditions that fostered key connections with peers were being abandoned by a new generation of Travellers. Others reflected on support systems and family networks being undermined as a result of what were seen as deserted rural communities. Some highlighted that an increased focus on technology and a faster and more individual way of life, was having a detrimental effect on connections within communities:

**“…the older generations learnt the social skills, the communication skills and the interactions of how to get on with people…” (Garret, Farming Official)**

Many of these participants argued that historically, community contact meant that fewer people were isolated and that there were more opportunities to give help. Against a backdrop of recession and rising unemployment however, it was felt that more vulnerable groups in particular were falling into isolation and being cut off from essential human contact. “Contact” therefore was seen as encapsulating both a literal (proximity, communication) and metaphorical (connection and belonging) significance in men’s lives:

**“Men need to belong in relationships, in the workplace, in the family, in schools and in the community.” (Alice, Teacher).**

Sustaining or staying “in contact” with men during times of psychological distress was seen as simultaneously facilitating opportunities for help-seeking and help-giving. Indeed, many reflected on a gendered dimension to contact. Notably, the value of “just being there” did not necessarily have to translate into “doing” or “saying” anything, but afforded more informal and subtle opportunities for a type of organic ebb and flow of help/support to be sought, given and received. Potential help-givers reflected on the importance of maintaining an intuitive “raised antennae,” and of being “open and available.”

Given the right conditions—contact, alertness, and openness – many participants felt that men in psychological distress would be more favorably disposed to asking for and accepting help. Responding in a “contactful” and prompt way was seen as particularly important for men as it was felt that men succumb to the negative effects of depression faster than women, and need to be tended to quickly once they start to feel in psychological distress to prevent the slippery slide to suicide or more serious psychological distress:

**“If a man is depressed…he goes down much quicker than a woman.” (Mildred, Public Health Nurse)**

### “Navigating Roles, Responsibilities, and Boundaries to Support Men in Psychological Distress”

The issue of “roles” in supporting someone in psychological distress and the responsibilities and boundaries attached to such roles, emerged as a highly contentious finding in this study. This was particularly pronounced where psychological distress was perceived to be gravitating towards being “at risk” of suicide.

Against a backdrop of what were perceived to be varying degrees of competency in fulfilling such roles and acting on one’s “duty of care” to others, most participants felt that suicidal behavior could be anticipated or predicted in advance by being more knowledgeable or skilled in recognizing particular signs of psychological distress associated with increased suicide risk, or “reading the signs.” Signs were generally perceived as more obvious physical manifestations of psychological distress, with crying in particular noted as one of the signs on which participants would act. The majority however acknowledged the problematic nature of predicting suicidal behavior in particular, of reading “signs” and knowing “when” to intervene due to uncertainty about when it was appropriate to intervene and whether the offer of help would be welcome or not:

**“…you don’t know whether he’s grand or not grand…” (Alan, Men with a History of Substance Misuse).**

CG participants, in particular, felt torn between wanting to be more aware of “signs” and “at risk groups” of suicide, while recognizing that such an approach could result in missing out on more subtle or less obvious “signs”:

**“I’d be afraid of almost categorizing and assuming that somebody might be suicidal and I might actually miss the person who is…its very random really.” (Sr. Bridget, Religious Group).**

The notion of a “not one solution fits all” approach to supporting someone in psychological distress ran parallel with the perceived requirement for different skillsets in different circumstances in terms of supporting those in psychological distress.

While there was repeated reference to the importance of intuition and natural instinct, most participants felt that having the requisite skills and undertaking appropriate training were essential to recognize and to respond to “signs” of psychological distress:

**“The more knowledge and the more training and involvement you have the more likely you are to give help.” (Kieran, Farmer)**

Many expressed concerns about “failing” or being rejected as a help-giver after making an intervention with someone in psychological distress. This fueled the belief that “professionals” had the requisite “skills” and “tools” and were therefore best placed to intervene. The majority of participants felt that certain roles—aligned with particular skillsets (doctor, nurse, therapist) or with positions of responsibility with regard to others (teacher, coach)—endorsed or legitimized help-giving:

**“…I think because you are in the profession, people would come to you—‘you’re a nurse can you help me?’…” (Jane, Public Health Nurse).**

Nevertheless, even within these professional roles, there was still ambiguity in relation to what constituted the boundaries of “duty of care” to others. Indeed, there was repeated references to a “burden of responsibility” as CG participants, in particular, grappled with the grey area of role expectations against a backdrop of varying degrees of perceived competency in fulfilling such roles:

**“…there is a fear there as well that if a child dies by suicide and the issue of bullying comes up, they [family] want to find a reason why…all of a sudden it is ‘teachers need to be more aware’.” (Alice, Teacher)**

The issue of boundaries also emerged as a concern for family members of those in psychological distress. Notwithstanding their proximity to potentially notice and act on “signs,” it was of concern to many that “untrained” or “unskilled” family members might be out of their depth in terms of offering the “right” support. There was also concern about family members being “too close” and potentially missing something out of the ordinary:

**“…my biggest fear I think would be that I wouldn’t cop it…you don’t see it within your own family…not seeing what’s in front of my nose.” (Tracey, Trave**l**ler)**

In the absence of what were perceived as the requisite skills or training, many participants felt overwhelmed by the prospect of offering help or paralyzed by the fear of doing “the wrong thing” and potentially compounding the problem further:

**“…you might just say the wrong thing or send them in the wrong direction…if that person is dead the next morning then I didn’t help did I?” (Frank, Farmer)**

Indeed, many felt that the prudent option was often to “take a step back” so as not to risk misreading “signs.” The majority of participants spoke about feeling more comfortable and more confident offering help if they knew their boundaries and when and how to be a conduit to other (“closer,” more professional or more qualified) help-givers or support services who might be better placed to help.

Help-giving was linked not just to how predisposed the help-giver was to giving help but to how receptive someone in psychological distress might be to asking for or accepting help. There was particular concern about potentially over stepping a boundary by seeking help from a loved one. The perception was that, once such a boundary was crossed, “the cat was then out of the bag” and there was no going back and no escape from the pressure cauldron that such disclosure could potentially cause in an intimate family relationship:

**“…you don’t want them [parents] to know everything…if you tell them something, you’re wondering what are they thinking. Whereas with your friends, you’re not with them as often so you get that release from it” (Michael, Young Men)**,

## Discussion

The aim of this study was to investigate how masculine norms impact men’s help-seeking as well as care givers behaviors and willingness to support men in need of psychological help or perceived to be at risk of suicide. The literature to date has gravitated towards a now familiar binary argument—men are largely “the problem” when it comes to problems with their mental health (emotionally inept, resistant to seek help) and service providers do not know how to reach out to men (Grace et al., 2016). The findings from this study underline the futility of this more simplistic and reductionist approach and provide the basis for (a) looking beyond at risk population groups for male suicide to more carefully examine the sociocultural contexts and environments of men’s lives that mediate such risk, and (b) embracing tensions and complexities in relation to how men ask for and accept help and how competent and confident potential help-givers might feel about supporting men in psychological distress.

Reflecting upon the wider context of men and mental health, participants in this study emphasized the importance of men having a sense of connection and belonging in their lives. Against a backdrop of economic recession and what was seen as an erosion and disintegration of more disadvantaged and rural communities, and disruption of family and community networks, participants identified a number of triggers (e.g., unemployment) and transition points (e.g., relationship difficulties) that potentially pushed men into isolation. The impact on men’s lives was an undermining of their masculine and role identities and an erosion of their sense of place, autonomy and control. Links between recession/rising unemployment and increasing suicide rates are well established (Dillon & Butler, 2011; White et al., 2011) but few studies have elaborated on the exact nature of this relationship.

Durkheim’s theory of anomic suicide (Durkheim, 1897) provides insight to the sense of disillusionment associated with a loss of moral compass and social direction, which more typically occurs during recessionary periods or social disruption. More recent theories of suicide such as Joiner’s Interpersonal-Psychological Theory of Suicidal Behavior (Joiner, 2005) is also consistent with these findings and aptly captures the wider context of men, help-seeking and suicide. Joiner theorized that people die by suicide because they have both the desire and the means to do so. He identified three constructs which are central to suicidal behavior—thwarted belongingness and perceived burdensomeness (which are related to suicidal desire) and acquired capability for suicide (related to means). Direct links can be made with the concept of masculinities in that there is a common link between unemployment, family status and physical illness and developing perceptions of burdensomeness and feeling that they are making matters worse for the people in their lives (Van Orden, 2010).

Previous studies have reported that in the face of isolation and disconnection, and without genuine connection and mutual understanding with potential help-givers, men are unlikely to talk openly about or seek help for their depression (Berger et al., 2012). Staying plugged in or “in contact” with key societal institutions—such as family, community, work, and sport—was seen as providing a bedrock of routine, ritual and contact in men’s lives, acting as a buffer against disruption, isolation and disconnection, as well as simultaneously preserving masculine identity through a sense of belonging. For some, simply being “in contact” and engaging in routine conversations or shared activities with other men was sufficient, a finding that is consistent with Thompson and Whearty’s (2004) notion of “covert intimacy.”

In contrast to more traditional or dominant constructions of masculinity – which emphasize competitiveness, self-reliance and lack of emotional expression – this discourse of genuine connection highlights opportunities for men to make and sustain contact and a sense of genuine connection with others. This also raises interesting questions about whether men are being offered the “right” help during times of psychological distress. It may not so much be a question of men being resistant to help-giving for mental health issues but rather having varying degrees of ambivalence depending on the type of help available and the context in which it is being offered (Addis, 2011). These findings serve as an important backdrop to discourses on “at risk” groups for male suicide and point towards the need for an increased focus on at risk sociocultural contexts that shape whether men are likely to ask for, be offered or accept help during times of psychological distress.

Many tensions and complexities emerged in relation to how men in psychological distress ask for, are offered or accept help. An array of factors shaped men’s willingness to accept or “come out” with something that is affecting their mental health, as well as potential help-givers perceived capacity to give help. Consistent with previous findings, men who adhered to more traditional masculine characteristics or norms equated being in psychological distress with failure and a weakened masculine identity (Mahalik, Burns, & Syzdek, 2007), while reaching a certain threshold of psychological distress was a barometer used by both help-seekers and potential help-givers to gauge when to ask for or give help (Johnson et al., 2012, Thompson, Hunt, & Issakidis, 2004).

The notion of a “double burden,” or “double jeopardy” (Good & Wood, 1995; Levant et al., 2009; Houle et al., 2008) encapsulated men’s sense of shame in asking for help as compounding their “failure” to manage their own problems. Nevertheless, reluctance to ask for help did not rule out openness to the offer of help (Strike et al., 2006). For (potential) help-givers, it was important to remain open, available and to keep in contact with someone who was psychologically distressed, even if the opportunity to approach that person had not yet arisen. Indeed “just being around” was seen as granting an opportunity for (potential) help-givers to maintain a “raised antennae,” and didn’t have to necessarily translate into doing or saying anything (or being an expert). It was equally important to facilitate support in ways that did not undermine a man’s masculinity further so that he should be enabled to retain a sense of normality, autonomy and control (Mahalik et al., 2007) and not to be treated any differently as a man.

In response to the many barriers to help-giving identified above, these findings highlight the importance of gatekeeper training to enable these individuals to identify those at risk, assess the level of risk, manage the situation accordingly and ultimately to increase the number of interventions with those in distress (Gould & Kramer, 2001; Isaac et al., 2009).

The issue of trust emerged as being central to the triad of asking for, offering/being offered and accepting help. Given the right conditions—contact, openness, and trust—most participants felt that men in psychological distress would be favorably disposed to asking for and accepting help. While this placed family and friends as the preferred source of support for many, there was both a fear of placing undue worry on loved ones as well as adjusting to how such revelations might impact on the dynamic of intimate relationships. There was also a danger for help-givers of being too close and missing out on or not wanting to face up to “signs” of psychological distress in their loved ones (Gryglewicz, Elzy, Brown, Kutash, & Karver, 2014; Oldershaw, Richards, Simic, & Schmidt, 2008). Finally, the role of female spouses in caring for men and serving as their primary source of support has been well documented within the literature (Johnson et al., 2012, O’Brien, Hunt & Hart, 2005). However, there is evidence to suggests that this may be problematic because of the potentially negative effects that this can have for female spouses (Schrank et al., 2016; Well, First, it Out, & Training, 2013), as well as reproducing gender stereotypes of women’s role as caregivers (Lachance-Grzella & Bouchard, 2010).

Tensions and complexities also emerged in relation to roles, responsibilities and boundaries in offering or accepting help. Through various professional and voluntary roles, many male participants in particular, felt a sense of responsibility and a moral duty of care to others (Eagly, 2009). Nevertheless, participants also felt the need to be able to recognize “signs,” to be trained with the requisite knowledge and skills, and to know when and how to “step in” to adequately support an individual in psychological distress (Gryglewicz et al., 2014; Yap & Jorm, 2011).

This “burden of responsibility” gravitated between a sense of inertia associated with not doing anything to help, to a fear of doing the wrong thing and potentially compounding the problem further. The latter was seen as being exacerbated by what was seen as limited referral options, which has been reported to have a negative knock on effect on gatekeepers acting in their role of help-giving or assuming a “referral” role (Capp, Deane & Lambert, 2001). While this prompted many participants to assert that “experts” possessed the requisite “skills” and “tools” to support someone in psychological distress, this is at odds with a body of literature that identifies gatekeeper training as having a crucial role in identifying those at risk (Gould & Kramer, 2001). Whist designated groups (doctors, nurses, social workers) have more training and skills than community members, evidence points to the merits of training those in prime settings (such as teachers or coaches; Cerel et al., 2012) as well as family and friends

(Cerel et al., 2012; Cross et al., 2007, 2011) who are strategically placed to offer help. These findings have important implications for suicide prevention policy and practice. Increased outreach and training efforts are needed to challenge myths associated with being able to read “signs,” being able to predict suicide or not being expert enough to intervene.

Paradoxically, help-givers’ concerns about not being “expert” enough to give help were at odds with what were perceived as help-seekers more basic and fundamental needs; simple contact, cups of tea or activity based support. This is mirrored in recent literature, which identified that men derived support from activity-based practical things and valued belonging and being part of a community of other men (Lefkowich & Richardson, 2016; Lefkowich, Richardson, & Robertson., 2015; Shaw, Gullifer, & Shaw, 2014). Likewise, while there was a perception that suicidal behavior could be anticipated in advance by being more knowledgeable or skilled in reading “signs,” it is well documented that warning signs for suicidal behavior may not always be present or may be difficult to interpret (Wasserman, Durkee & Wasserman, 2009). Although it was felt that many men struggle with finding the right language to express emotions (or simply that “men don’t talk”), it was also apparent that there were many nuances and subtleties in men’s use of language that often went under the radar.

These findings highlight the tensions that exist between potential goodwill and good intentions on the part of help-givers on the one hand, and the many challenges and fears that stand in the way of help being given, on the other. It is worth considering whether accounting for these issues might support help-givers to feel more competent and confident in giving help, thereby facilitating improved help-seeking and help-taking opportunities among those in psychological distress.

### Limitations

This study had a number of limitations. First, while the sampling strategy endeavored to be inclusive of diverse population groups, it cannot purport to be representative of all “HRS” and CG groups. Second, some CG participants had more limited experience than others of engaging men in the past. While this, somewhat ironically, epitomizes and reinforces some of the study findings, it presented some participants with a narrower frame of reference from which to contribute to focus group discussions. Third, as the study findings relied on participants’ recollection of past experiences, it was assumed that such recollections and perspectives were both accurate and honest. Findings may have been enriched, for example, by the inclusion of participants with more direct experience of mental health issues or exposure to suicide. However, recruiting such participants would have been challenging both ethically and practically. Fourth, getting access to some key service providers such as GPs and clinical psychologists proved to be particularly difficult, and this may have compromised the generalizability of the study findings. Finally, while all participants were assured of confidentiality and invited to speak candidly, it is possible that some may have been guarded in sharing personal experiences out of fear of compromising family members/loved ones or colleagues.

## Conclusion

The findings from this study confirm that the starting point for male suicide prevention strategies needs to be in tackling the root causes of men’s disconnection from key societal institutions such as family, education, and community, and on building capacity particularly within more marginalized communities of men. The findings also highlight how masculine norms impact men’s help-seeking as well as care givers willingness to support men during times of psychological distress, by identifying situations and opportunities where men may be open to seeking help, from whom and in what circumstances they may be most likely to accept help, and what the key barriers and enabling factors are likely to be in influencing those who may be in a position to offer help. Thus, a key basis for reaching men with effective suicide prevention interventions is to look beyond men’s help-seeking as a passive, one-dimensional construct, to a more dynamic triad of help-seeking/giving/taking behaviors that intersect in multiple and complex ways and that are embedded in the sociocultural context of men’s lives.

Rather than being seen as confusing or muddying the waters in terms of approaches to suicide prevention, these findings highlight the importance of not falling into the trap of stereotypical or essentialist notions of “what works” in relation to how men ask for, are offered or accept help. In addition to having informed a gendered approach to suicide prevention within Ireland, the study findings have implications for the wider field of gender, mental health and suicide prevention, and transcend the Irish context.
